# The sulphated polysaccharides extract ulvans from *Ulva armoricana* limits Marek’s disease virus dissemination in vitro and promotes viral reactivation in lymphoid cells

**DOI:** 10.1186/s12917-022-03247-y

**Published:** 2022-04-27

**Authors:** Frédérick Bussy, Sylvie Rémy, Matthieu Le Goff, Pi Nyvall Collén, Laëtitia Trapp-Fragnet

**Affiliations:** 1 Amadeite SAS, 56580 Bréhan, France; 2 Olmix, SALe Lintan, 56580 Bréhan, France; 3 INRAE, Université de Tours, ISP, F-37380 Nouzilly, France

**Keywords:** Marek’s disease virus, Seaweed, *Ulva armoricana*, Searup®, Cytotoxicity, Viral replication, Viral dissemination, Viral reactivation

## Abstract

**Background:**

Marek’s disease (MD) is a highly contagious lymphoproliferative disease of chickens caused by an alphaherpesvirus, Marek’s disease virus (MDV). MD is presently controlled by systematic vaccination of animals, which protects efficiently against the development of clinical disease. However, MDV vaccines do not prevent the multiplication and spread of MDV field strains and may favor the emergence of strains with increased virulence. Therefore, MDV persists to be a major problem for the poultry industry and the development of new alternative strategies to control MDV is needed. Seaweed extracts have previously been shown to exert immunomodulatory and antiviral activities, especially against herpesviruses. The objective of the present study was to explore the effect of *Ulva armoricana* extracts on MDV infection in vitro.

**Results:**

We could demonstrate that the ulvan extract as well as its vitamin-enriched formulation reduce the viral load by about 80% at 24 h post-infection in infected chicken fibroblasts at concentrations that are innocuous for the cells. We also observed a substantial decrease in MDV plaque size suggesting that ulvans impede MDV cell-to-cell spread in vitro. Moreover, we showed that ulvan extract could promote MDV reactivation in lymphoid cells.

**Conclusions:**

Our data provide the first evidence that the use of the ulvan extract could be a good alternative to limit MDV infection in poultry.

## Background

Marek’s disease virus (MDV) is an alphaherpesvirus inducing lethal lymphoma in chickens. MDV infection progresses in 3 major phases: (i) an early cytolytic infection corresponding to the entry of the virus through the respiratory tract and its transport to the lymphoid organs where it infects B-cells and activated T-lymphocytes; (ii) a latent phase (between 7 to 10 days post-infection (pi)) in CD4 + T lymphocytes, which is characterized by the integration of the viral genome into the genome of the host cells; (iii) the tumor phase occurring in CD4 + T lymphocytes leading to malignant lymphoma formation and the death of animals from the 3rd week of infection [1, 2]. Concomitantly, a late cytolytic phase resulting from MDV reactivation in latently-infected lymphocytes occurs. The capacity of MDV to reactivate constitutes a perennial source of virus production and contributes to the spread of the virus to the skin, more particularly to the cells of the feather follicle (the only site of production and excretion of free infectious viral particles).

MDV is endemic worldwide and, despite the availability of vaccines, MDV continues to threaten the poultry industry with economic losses estimated at 1–2 billion dollars per year [3, 4]. Vaccination protects infected animals against the development of tumors induced by MDV, but does not prevent the replication and the shedding of the virus in poultry flocks [5]. In addition, the vast vaccination campaigns implemented since the end of the 1960s have caused the virus to gradually evolve towards higher pathogenicity allowing the virus to overcome the protection provided by current vaccines [6–8]. To date, no treatment (other than vaccine prophylaxis) is available to control the replication of the virus and its dissemination in the environment. Consequently, the development of alternative and sustainable strategies to control MDV is needed.

Numerous scientific publications have reported that algae extracts or algae-derived compounds can exhibit inhibitory activities against viral infections (for reviews [9, 10]). The first study demonstrating the anti-viral activity of seaweed extracts against herpesvirus infections was reported in 1974 [11]. Since then, many active compounds in algae have been shown to interfere with the viral life cycle of alphaherpesviruses Herpes Simplex virus type 1 and 2, the betaherpesvirus Human Cytomegalovirus and the gammaherpesviruses Human Herpesvirus type 6 and 7 [12–25]. Algae polysaccharides can exert their anti-viral activities by preventing the entry and multiplication of the virus in its target cell, and/or by modulating the immune response [10].

While research on the antiviral and immunomodulatory properties of algae is sparking great interest in human medicine, studies related to animal health are still scarce, particularly with regard to herpesvirus infections. We recently demonstrated that ulvans, the sulfated polysaccharides (SPs) fraction purified from *Ulva armoricana*, is able to activate chicken heterophils and monocytes in vitro and in vivo [26]. This suggested that ulvans can potentially be used as an immunostimulant for controlling infectious diseases. Here, we explored whether the ulvans fraction and its formulated version, dubbed and commercialised as Searup® could reduce MDV lytic replication and MDV reactivation from latently infected cells in vitro*.*

## Results

### The ulvans SPs fraction does not alter the viability of avian cells

Prior to determining the effect of ulvans and Searup® on MDV replication, we evaluated the cytotoxicity of the extracts on avian cells. Chicken embryo fibroblasts (CEFs) and the lymphoid cell line 3867 K were treated with four increasing doses (0.5; 1; 1.5 and 2 ml/l) of the vitamin complex (VitComp), Searup® or the ulvans extract. Untreated cells were maintained in culture and used as negative control. In order to exclude any side effects induced by degradation products of the compounds and/or cellular metabolites, the culture media was replaced after 48 h by fresh media supplemented (or not for the control condition) with the defined treatment doses. At 24, 48, 72 and 96 h post-treatment, the viability of CEFs was monitored by evaluating the level of ATP produced by the cells (CellTiter Glo assay) and the viability of 3867 K was assessed by flow cytometry using the Viability Dye eFluor™ 780.

**Fig. 1:**
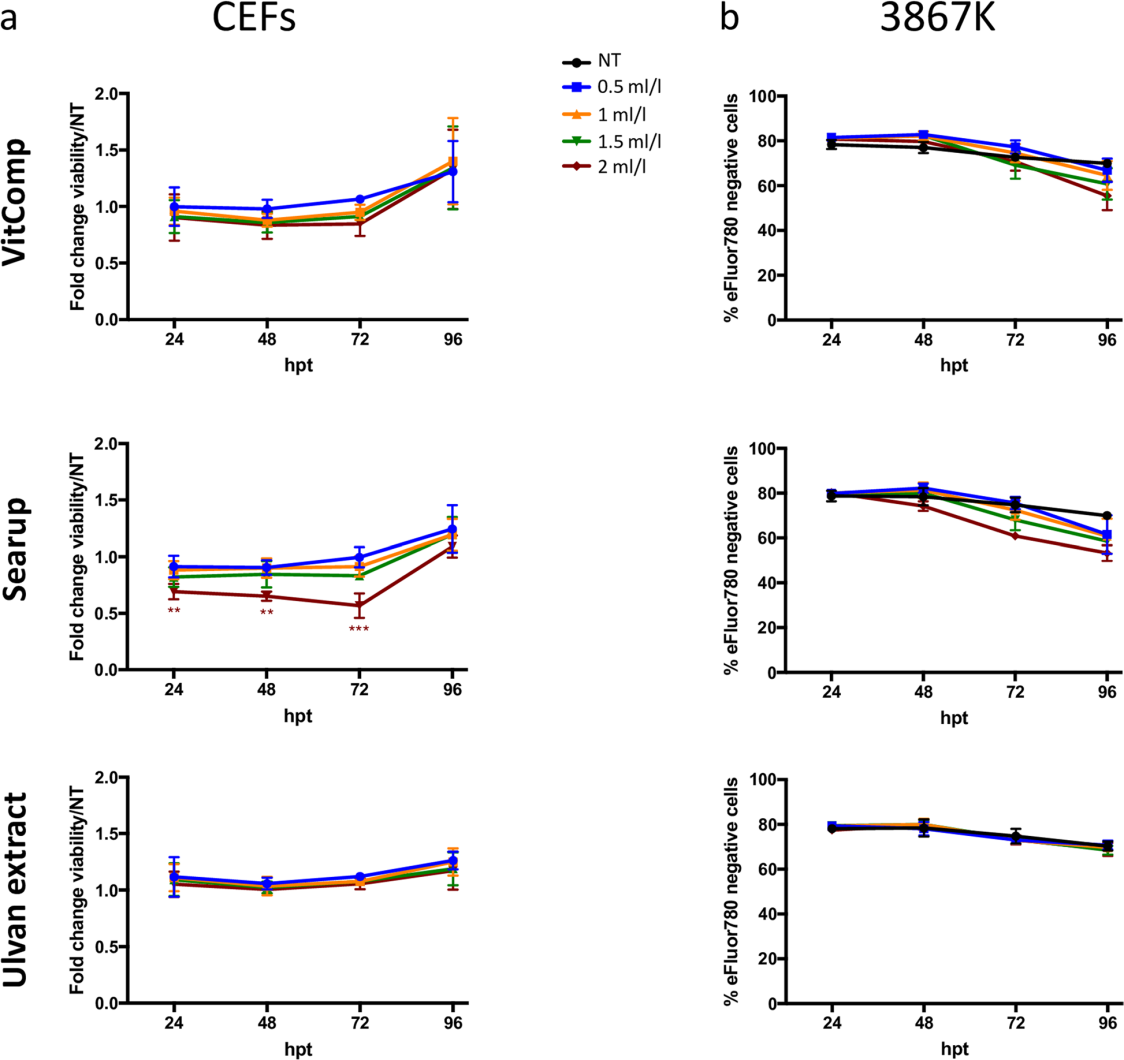
Ulvans does not affect avian cell viability. Chicken embryo fibroblasts (CEFs) and MDV transformed T-lymphocytes (3867 K) were treated with vitamin complex (VitComp), Searup® or the ulvans extract at the indicated concentrations or left untreated (NT). At indicated time points (hpt), (A) the viability of the CEFs cells was assessed using the CellTiter-Glo® kit. Results are presented as fold-change of ATP production values assessed for treated cells relative to ATP produced in non-treated cells (NT = 1). (B) The viability of 3867 K cells was estimated by flow cytometry using the Fixable Viability Dye eFluor™ 780. Three independent experiments are represented as means (+/− SEM) of the percentage of viable 3867 K cells (eFluor negative). Statistics analyses did not show any difference (*P* > 0.05)

We could show that the purified ulvans extract and the vitamin complex had virtually no cytotoxic effect on primary chicken fibroblasts (Fig. 1A). Treatment with the highest concentration (2 ml/l) of Searup® induced a mild decrease of viability of the CEFs between 24 to 72 hpt, but no cytotoxicity was observed at lower doses. This effect of high-dose Searup® treatment on the metabolic activity of CEFs (ATP production) appeared to be reversible since the cells recovered at 96 hpt and may instead reflect a delay in cell proliferation.

The MDV transformed T-lymphocytes (3867 K) treated with the different formulations showed a level of viability similar to untreated cells (of about 80%), independently of the doses used (Fig. 1B). A slight decrease in the viability of 3867 K (albeit not statistically significant) was detected from 72 h after treatment with Searup® and the vitamin complex. However, this effect of the Searup® seems to be mainly associated with the vitamin complex contained in the formulated Searup® since the pure SPs extract (ulvans) was completely innocuous for the 3867 K cells. Consequently, our results indicate that the various treatments and in particular the ulvans extract showed a very limited cytotoxicity on avian lymphoid and fibroblast cells.

### Ulvans extract reduces MDV dissemination in vitro

**Fig. 2:**
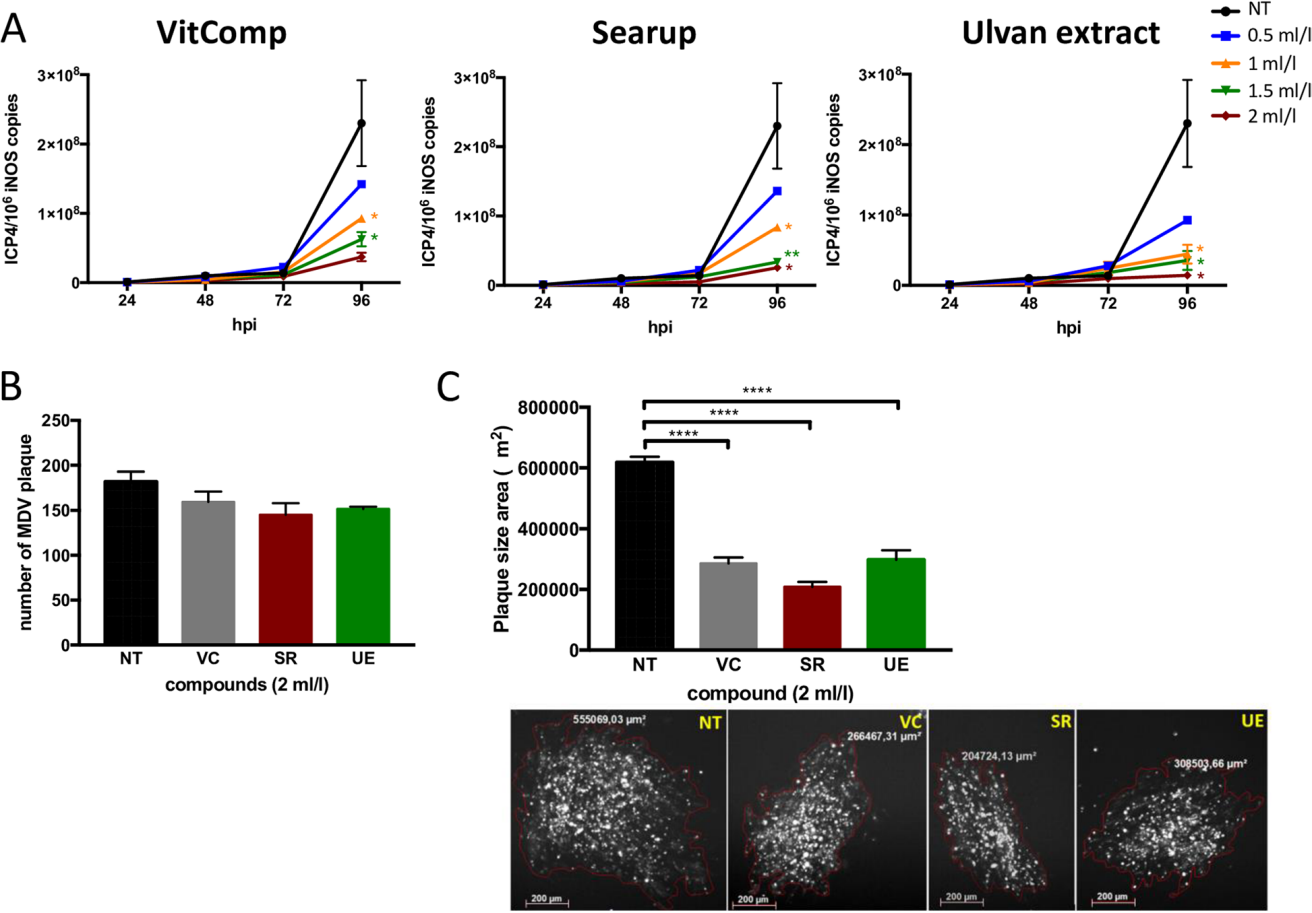
Ulvans reduces MDV lytic replication in vitro. CEFs were infected with the recombinant recEGFPVP22 virus and treated at 1 hpi with increasing dose (0.5, 1, 1.5 and 2 ml/l) of vitamin complex (VitComp), Searup® or ulvans. Infected non-treated CEFs (NT) were used as negative control. (A) At indicated time points (hpi), DNA was extracted and MDV replication was assessed by qPCR. The number of MDV genome copies (corresponding to the *ICP4* copy number) was normalized to 10^6^ cells (estimated by the *iNOS* copy number). (B) At 96 hpi, the number of MDV plaques was counted and (C) the average plaque size was measured from at least 50 plaques. Representative pictures of viral plaques observed upon 96 h of the different treatments are shown with their respective size (Bars, 200 μm). Data of three independent experiments are represented as means +/− SEM. * *p* < 0.05; ** *p* < 0.005; **** *p* < 0.0001

To determine whether ulvans treatment can impact MDV lytic replication, we infected CEFs and treated the infected cells with increasing doses of the vitamin complex, Searup® and the ulvans extract. The viral load was quantified at 24, 48, 72 and 96 hpi by qPCR. Our results showed that all treatments significantly decreased MDV lytic replication in CEFs (Fig. 2A) in a dose-dependent fashion with the strongest effect observed with concentrations from 1 ml/l. From 24 hpi, the viral load is reduced by about 80% following the treatment with Searup® and the ulvans extract at a concentration of 2 ml/l (the recommended dose in farms corresponding to the administration of 1 ml/l/24 h). This decrease is then maintained over time and reaches 89 and 94% at 96 hpi, respectively. To determine whether this reduced viral load was related to an activity of the ulvans on the replication of the virus or on its cell-to-cell spread, we counted and measured the plaques of MDV infection obtained after the treatments. We showed that the number of MDV plaques did not differ upon treatment (Fig. 2B). However, we observed a reduction of 2- and 3-fold in the size of MDV plaque upon treatment with the ulvans extract and Searup®, respectively (Fig. 2C). These results showed that the SPs fraction of the purified from *Ulva armoricana* as well as the commercialized Searup® formulation can impede MDV dissemination in vitro in the concentration range recommended in poultry farms (1 ml/l/24 h).

### Ulvans promotes viral reactivation in MDV transformed lymphoid cells

**Fig. 3:**
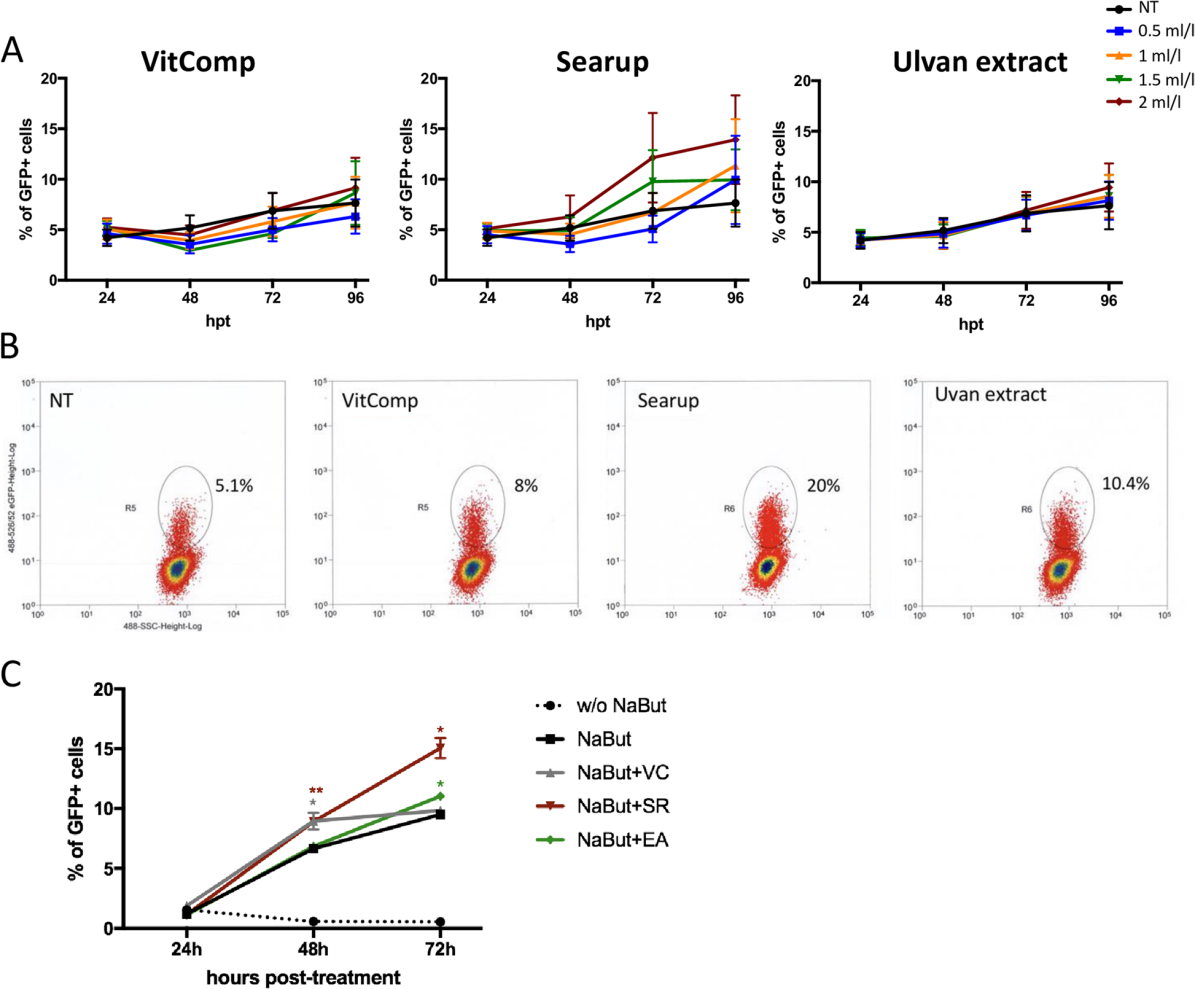
Ulvans promote MDV reactivation. (A) MDV transformed T-lymphocytes (3867 K) were treated with vitamin complex (VitComp), Searup® or purified ulvan extract at indicated concentrations or left untreated (NT). At 24, 48, 72 and 96 hpt, cells were stained with the Fixable Viability Dye eFluor™ 780. The percentage of cells in which MDV reactivates from latency (UL47-GFP positive cells) was then estimated by flow cytometry within the viable cell population (eFluor negative). Data of three independent experiments are represented as means +/− SEM. Statistics analyses did not show any difference (*P* > 0.05). (B) Representative cytometry dot plots obtained after 96 h of culture without treatment (NT) or with 2 ml/l of compounds. (C) The 3867 K cells were treated with VitComp, Searup® or ulvan extract (at 2 ml/l) in combination with an inducer of viral reactivation, the sodium butyrate (NaBut) used at 0.5 mM. Viral reactivation was estimated by flow cytometry within the viable cell population at 24, 48 and 72 hpt. Data of three independent experiments are represented as means +/− SEM. * *p* < 0.05; ** *p* < 0.005

We assessed whether the two ulvans formulations and the VitComp control could have an effect on the maintenance of MDV latency and viral reactivation (corresponding to the late cytolytic phase of infection) in vitro. To this end, we used the 3867 K cells, a lymphoblastoid cell line that expresses the green fluorescent protein (GFP) fused to the tegument protein UL47 upon reactivation, as described previously [27, 28]. The 3867 K cells were treated with the vitamin complex, Searup® and ulvans at different concentrations and the rate of viral reactivation (GFP positive cells within the viable cell population) was evaluated by cytometry upon 96 h of treatment (Fig. 3A and B). In cells treated with the vitamin complex or the ulvan extract, we observed that the proportion of cells in which the virus reactivated (GFP positive cells) increased similarly to the untreated cells from 24 to 96 hpt, independent of the doses used in the assay. At 96 hpt, we detected 7.6, 8.6 and 9.4% of GFP positive cells among the untreated cells, the cells treated with VitComp and the cells treated with the ulvan extract, respectively. Albeit not statistically different, the Searup® tended to induce an increase in viral reactivation since we observed 14% of GFP positive cells after 96 h of treatment at the highest dose (2 ml/l). We then investigated further the effect of the ulvans on viral latency/reactivation by treating the 3867 K cells with the VitComp, Searup® or ulvan extract (at 2 ml/l) in combination with sodium butyrate (NaBut 0.5 mM), a histone deacetylase inhibitor known to induce herpesvirus reactivation. The viral reactivation was assessed by cytometry at 24, 48 and 72 hpt (Fig. 3C). As expected, the NaBut treatment substantially increased the proportion of cells in which the MDV reactivated, reaching 9.5% of GFP positive cells at 72 hpt, while in untreated cells only 1.5% were GFP positive. This increase in viral reactivation is also detected upon co-treatment of the cells with NaBut together with the VitComp, Searup® or Ulvan extract. The level of reactivation in cells treated with NaBut and the vitamin complex is comparable to the reactivation in cells treated with NaBut treatment alone, despite a slight transitory increase at 48 hpt. The ulvan extract showed only a slight effect on the NaBut-induced reactivation at 72 hpt (11% of GFP positive cells). However, in cells treated with Searup® we could clearly observed a higher proportion of cells in which the virus reactivated with 8.9 and 15% of GFP positive cells at 48 and 72 hpt, respectively. These results suggest that ulvans did not prevent a chemically-induced reactivation but that the ulvans fraction formulated in the Searup® version might rather promote MDV reactivation.

## Discussion

The intensification of chicken farms around the world, the incomplete protection of anti-MDV vaccines and the emergence of new hypervirulent pathotypes of MDV suggest that MDV could become a major problem in the poultry industry if new therapies or vaccines are not developed. Most efforts to control MDV are focused on developing next-generation vaccines. Although many natural products or chemicals have been shown to be effective against many viral infections affecting humans and animals, only a few studies have reported antiviral activity of compounds against MDV infection [29–32]. Here, we have shown that ulvan extract can significantly reduce the replication and dissemination of MDV in vitro with a limited cytotoxicity in avian lymphoid cells.

Several studies reported that algae SPs are able to directly inhibit transcription/replication of alpha and gammaherpesviruses after viral internalization in the host cell [11–13, 15, 16, 18–20, 24, 33]. Our data demonstrate that *Ulva armoricana* SPs also limit MDV replication in chicken fibroblasts since the viral load was strongly reduced while the number of MDV plaques was similar to that of untreated cells. In addition, the significant reduction of MDV plaque size suggests that the ulvans extract prevents MDV spread. Algae SPs have also been shown to be able to hinder the early stages of viral infection (adsorption/attachment and penetration of the virus) by binding directly to viruses or to viral receptors present on the surface of cells [16, 22, 23, 34–37]. MDV is strictly associated to cells and no extracellular viral particles are produced in vitro. Therefore, ulvans SPs cannot exert a direct neutralizing activity on MDV virions but might rather have an effect on cell permissiveness to MDV infection, which in turn reduces MDV spread by cell-to-cell fusion. Previous reports indeed showed that SPs from different sources could be powerful inhibitors of viral cell-cell spread and/or fusion [34, 38–40]. Also, in our previous study we have found that ulvan extract of *Ulva armoricana* can modulate the immune response notably by triggering the expression of a number of pro-inflammatory cytokines (e.g. IL-1β, IFN-α and IFN-γ) [26]. We thus cannot exclude that *Ulva armoricana* SPs extract can also control MDV infection/dissemination through the secretion of factors such as IFN-α which was shown to strongly impede the infectivity of MDV [41].

Aside the effect of the ulvan extract, we showed that the vitamin complex alone also reduced the replication of MDV. Nutrients, notably vitamins A and D, are known to exert antiviral activities and to stimulate immunity (for review [42]). Likewise, even though the vitamins contained in the algae formulation are low-concentrated, we cannot exclude that they may potentiate the effect of the ulvans extract.

In latently infected lymphoid cells, we observed that treatments with ulvans have a limited effect on viral reactivation when used alone. However, in combination with a reactivation-inducing chemical treatment (sodium butyrate), SPs extract from *Ulva* and especially its formulated version Searup® could promote the reactivation of MDV. We have not currently identified the mechanisms/factors triggered by ulvans that may contribute to MDV reactivation. However, among the many mechanisms identified to induce herpesvirus reactivation, we suspect that ulvans may promote MDV reactivation through the induction of pro-inflammatory cytokines expression, oxidative stress and/or the modulation of key cellular pathways (for review [43]). Interestingly, we previously demonstrated that ulvan extract of *Ulva armoricana* can activate heterophils and monocytes via the activation of Toll-Like receptor 2 and 4, resulting in the release of pro-inflammatory cytokines, including IFN-γ, which is a potent inducer of herpesvirus reactivation [26, 44]. Also in the case of Epstein Barr latent infection, it was shown that lytic cycle can be induced through TLR2 and B-cell receptor activation [45]. In addition, ulvans induced a strong oxidative burst in heterophils and monocytes accompanied by a significant secretion of nitric oxide (NO). Oxidative stress and reactive oxygen species are powerful inducers of herpesvirus reactivation, as we have shown in particular for MDV [46, 47]. Finally, ulvans may promote the re-entry into lytic cycle by modulating several cellular pathways involving notably protein kinase C, p38MAPK, c- Jun N-terminal kinase (JNK), ERK kinase and PI3 kinase, which have been identified to trigger herpesvirus reactivation [43, 48].

In conclusion, we demonstrated that the *Ulva armoricana* SPs extract ulvans and its commercialized Searup® formulation considerably reduce the dissemination of MDV in vitro with limited to no cytotoxicity for avian cells. Clearly the anti-MDV activities of ulvans warrant further investigation in vivo, however, based on our data it can be speculated that the ulvans SPs extract could limit MDV infection at the early stages of infection. Ulvans could indeed exert an antiviral activity by limiting replication/dissemination of MDV and/or by stimulating the innate immune system of chickens [26]. In addition, at a later time of infection, treatment of chickens with ulvans could promote MDV secondary cytolytic infection (viral reactivation) occurring in MDV transformed T-cells. The resulting expression of MDV antigens and viral production could thus help to “purge” the cells latently infected either by the immune system or by virus-associated toxicity.

## Conclusion

Altogether, our results identify ulvans as a natural seaweed extract with potent antiviral activity against MDV, and potentially other avian viruses, that could be readily exploited in alternative treatment strategies for controlling viral diseases in poultry farming.

## Methods

### Cells and virus

Chicken embryonic fibroblasts (CEFs) were prepared from 12-day-old specific pathogen-free White Leghorn (LD1) embryos and maintained in culture as previously described [49]. CEFs infection was carried out in P6-well plates with 225 plaque forming unit/well of the recEGFPVP22 recombinant MDV [50]. The 3867 K-lymphoblastoid cell line was established from a renal lymphoma induced by the highly pathogenic MDV clone vRB-1B 47EGFP and maintained in culture as previously described [27].

### Treatments

Cells were treated with increasing doses of (i) the *Ulva armoricana* SPs fraction (ulvans) (ii) Searup® (Olmix SA) composed of the ulvans fraction formulated with vitamins A and D3 and (iii) the vitamin solution without seaweed extracts (VitComp) containing vitamins A and D3 according to the European legislation (EU 2015/724 and EU 2017/1492). The range of concentration (0; 0.5; 1; 1.5 and 2 ml/l) of SPs extract from *Ulva* corresponds to 11,5 mg/L to 46 mg/L of active seaweed ingredient. In the formulated version Searup®, the dose of SPs extract range between 8 mg/l to 32 mg/L. Of note, the concentrations used in the study was defined according to the recommended dose of Searup® (1 ml/l) used in poultry farms. Treatments were added to the culture media of infected CEFs from 1h post-infection (hpi). The 3867 K cells were cultured at a density of 5.10^5^ cells/P6-well plates in media containing the different treatments. All media supplemented with the different treatments doses were renewed after 48 h of culture. In a second set of experiment, the 3867 K cells were treated with 2 ml/l of VitComp, Searup® or ulvans extract in combination with 0.5 mM of sodium butyrate (NaBut; Sigma-Aldrich), a histone deacetylase inhibitor known to trigger herpesvirus reactivation [28, 51, 52]. Cells were treated for 24 to 96 h depending on the test performed. All media supplemented with the different treatments doses were renewed after 48 h of culture.

### Cell viability assays

The viability of CEFs incubated with the different concentrations of VitComp, Searup® and ulvans was assessed by quantification of ATP levels using the CellTiter-Glo® kit (Promega) according to the manufacturer’s instructions. Cells (5 × 10^3^) were grown in opaque-walled 96-well plates (Thermo Scientific) in 100 μl complete media containing the different doses of VitComp, Searup® or ulvan extract. At 24, 48, 72 and 96 h post-treatment (hpt), cells were washed in PBS1X and incubated with 100 μl of ATP detection reagent for 10 min at room temperature in the dark. Luminescence was measured using a GloMax-Multi Detection System (Promega). Relative ATP production values for treated cells were calculated as fold changes with reference to mean values obtained for non-treated cells (set to 1). Three independent experiments were performed in triplicate.

The viability of 3867 K lymphoid cells was assessed at indicated time points using the Fixable Viability Dye eFluor™ 780 (eBioscience) according to standard procedures. Viable cells were detected by flow cytometry using a MoFlo AstriosEQ high-speed cell sorter (Beckman Coulter Inc., Brea, CA, USA). A first gating was based on morphological criteria (FSC/SSC) in order to eliminate cellular debris and damaged cells. A second gating was then performed based on the eFluor 780 staining which allowed us to discriminate between dead (eFluor positive) and viable (eFluor negative) cells.

### Detection of MDV replication

CEFs infected with the recEGFPVP22 virus were treated at 1 hpi with increasing doses (0.5, 1, 1.5 and 2 ml/l) of the different treatments. Infected CEFs left untreated were used as negative control. MDV replication was assessed by real-time quantitative PCR (qPCR) as previously described [53–55]. At 24, 48, 72 and 96 hpi, DNA was extracted using the QIAamp DNA minikit (Qiagen) according to the manufacturer’s instructions. MDV genome copy numbers were quantified by qPCR using primers and probes targeting the viral ICP4 gene and normalized to 10^6^ copies of the cellular genome quantified by detection of the *Gallus gallus* inducible nitric oxide synthase gene (*iNOS*).

In 3867 K cells, MDV reactivation (lytically infected cells) was detected by flow cytometry based on the expression of the late antigen UL47-GFP. First, viable cells were selected on morphological criteria (FSC/SSC) and the eFluor 780 staining. The eFluor 780-negative population (viable cells) was then further gated for UL47-GFP expression to determine the percentage of reactivated cells (eFluor 780 negative/UL47-GFP positive).

### Virus cell-to-cell spread by plaque size assay

At 96 hpi, CEFs infected with MDV and treated with the vitamin complex, Searup® and ulvans were fixed using 4% paraformaldehyde and MDV plaque numbers were counted. MDV dissemination was evaluated by measuring the size of plaques visualized by GFP-expression, as previously described [50]. At least 50 plaques were measured for each treatment condition.

### Statistical analyses

All graphs and statistics were performed using the GraphPad Prism software version 5.02 (San Diego, USA). Data are presented as means and standard error of the mean (±SEM) from at least 3 independent experiments. The Kruskal-Wallis test was used to compare differences in multiple groups and the Mann-Whitney (two-tailed) test was used to compare non-parametric variables between two groups. *p* values < 0.05 were considered statistically significant as indicated in the figure legends.

## Data Availability

The datasets used and/or analysed during the current study are available from the corresponding author on reasonable request.

## Abbreviations

MD: Marek’s disease
MDV: Marek’s disease virus
SPs: Sulfated polysaccharides
CEFs: Chicken embryo fibroblasts
GFP: Green fluorescent protein
